# Glial fibrillary acidic protein as a biomarker in severe traumatic brain injury patients: a prospective cohort study

**DOI:** 10.1186/s13054-015-1081-8

**Published:** 2015-10-12

**Authors:** Jin Lei, Guoyi Gao, Junfeng Feng, Yichao Jin, Chuanfang Wang, Qing Mao, Jiyao Jiang

**Affiliations:** Shanghai Institute of Head Trauma, Department of Neurosurgery, Renji Hospital, Shanghai Jiaotong University School of Medicine, 1630 Dongfang Road, Shanghai, 200127 China; Department of Neurosurgery, Renji Hospital, Shanghai Jiaotong University School of Medicine, 1630 Dongfang Road, Shanghai, 200127 China

## Abstract

**Introduction:**

Glial fibrillary acidic protein (GFAP) may serve as a serum marker of traumatic brain injury (TBI) that can be used to monitor biochemical changes in patients and gauge the response to treatment. However, the temporal profile of serum GFAP in the acute period of brain injury and the associated utility for outcome prediction has not been elucidated.

**Methods:**

We conducted a prospective longitudinal cohort study of consecutive severe TBI patients in a local tertiary neurotrauma center in Shanghai, China, between March 2011 and September 2014. All patients were monitored and managed with a standardized protocol with inclusion of hypothermia and other intensive care treatments. Serum specimens were collected on admission and then daily for the first 5 days. GFAP levels were measured using enzyme-linked immunosorbent assay techniques. Patient outcome was assessed at 6 months post injury with the Glasgow Outcome Scale and further grouped into death versus survival and unfavorable versus favorable.

**Results:**

A total of 67 patients were enrolled in the study. The mean time from injury to admission was 2.6 hours, and the median admission Glasgow Coma Scale score was 6. Compared with healthy subjects, patients with severe TBI had increased GFAP levels on admission and over the subsequent 5 days post injury. Serum GFAP levels showed a gradual reduction from admission to day 3, and then rebounded on day 4 when hypothermia was discontinued with slow rewarming. GFAP levels were significantly higher in patients who died or had an unfavorable outcome across all time points than in those who were alive or had a favorable outcome. Results of receiver operating characteristic curve analysis indicated that serum GFAP at each time point could predict neurological outcome at 6 months. The areas under the curve for GFAP on admission were 0.761 for death and 0.823 for unfavorable outcome, which were higher than those for clinical variables such as age, Glasgow Coma Scale score, and pupil reactions.

**Conclusions:**

Serum GFAP levels on admission and during the first 5 days of injury were increased in patients with severe TBI and were predictive of neurological outcome at 6 months.

## Introduction

The outcomes following severe traumatic brain injury (TBI) are still poor, despite the great advances achieved in surgical techniques and intensive care over the past decades. It is estimated that 39 % of severely brain-injured patients die from their injury and 60 % are left with poor outcome [1]. Accurate determination of the extent of primary brain damage and the ongoing secondary injury after severe TBI is critical for the establishment of neurological prognosis and the guidance of appropriate therapeutic interventions [2]. Clinical characteristics such as the Glasgow Coma Scale (GCS) and modern imaging techniques have markedly improved the early assessment and care of severe TBI. Nevertheless, there are still concerns regarding these tools, including inability to manifest the actual pathophysiological or morphological status in the injured brain or being prone to influence the use of sedatives, analgesics, or muscle relaxants in current management protocols [3–5].

At present, we have no means of successfully monitoring the biochemical responses in the brain following severe TBI. A set of brain-specific biomarkers, measurable from accessible biological fluids, were recently reported to have the ability to reflect the severity and progression of brain damage, and were used to measure the response to treatment and offer prognostic information for injured patients [6–9]. Accurate outcome prediction also allows the identification of appropriate candidates for risky therapeutic strategies, which could limit the unnecessary treatment for patients who would have little possibility of survival or a favorable outcome [10].

Historically, a wide range of brain damage markers have been examined in TBI patients. However, owing to the limited tissue specificity and other concerns, most markers, including neuro-specific enolase and S100B protein, were compromised in routine clinical use [11]. Glial fibrillary acidic protein (GFAP) was recently reported to have greater prognostic value than other biomarkers in TBI patients [12]. As a monomeric intermediate filament protein concentrated in the astroglial cytoskeleton, GFAP is specific to brain tissue and is not routinely found in peripheral blood circulation. However, GFAP is released after astrocyte death, making it an ideal candidate marker for brain injury patients [13]. Several studies have found that the serum levels of GFAP on admission were significantly increased in TBI patients, while a correlation between serum concentrations and the pathological types of brain damage and clinical outcomes were also reported [14–18]. However, the changes in serum GFAP over time and the associated predictive utility over the acute days post injury are largely unknown.

Thus, the aim of this study was to evaluate the time course of serum GFAP levels in the first several days of injury, and to determine whether serum GFAP during this period could predict death and unfavorable outcome at 6 months for severe TBI patients. Our findings may be useful for early risk stratification for research and care of TBI patients.

## Methods

### Study population

This was a prospective cohort study that examined adult severe TBI patients admitted to a local neurotrauma center (Shanghai Institute of Head Trauma) in Shanghai, China, from March 2011 to September 2014. The institute, located in the Department of Neurosurgery of a tertiary teaching hospital (Renji Hospital), admits more than 400 TBI patients per year and provides 24-hour neurosurgery facilities and specialized intensive care for all patients.

The patients were included in the study based on established criteria, including older than 18 years, an acute TBI less than 4 hours before admission, a GCS score of 3–8 after resuscitation, and abnormal head CT scan on admission. Exclusion criteria were severe combined injury, unstable vital signs after resuscitation such as systolic blood pressure less than 90 mmHg or oxygen saturation less than 94 %, and pregnant women. Additionally, a total of 135 healthy blood donors who had no prior reports of TBI or neurological diseases were enrolled as the control group, with serum samples collected and analyzed for comparison.

This study was approved by the ethics committee of Renji Hospital, Shanghai Jiaotong University School of Medicine, and written informed consent was obtained from the control subjects and each severely brain-injured patient’s legal authorized representative owing to their comatose state within 24 hours of being deemed eligible.

### Treatment protocol

In Renji Hospital, nearly all injured patients were transported by emergency medical services (EMS) professionals to the emergency department (ED) shortly after trauma without intubation or sedation. Thus, we were able to evaluate the patients without the interference of prehospital interventions. On their arrival at the ED, patients with indications of TBI were neurologically assessed, received a computed tomography (CT) scan of the head, and then were screened for enrollment in this study. Large intracranial hematomas and hemorrhagic contusions, once confirmed by head CT, were evacuated immediately and the patients then transferred into the neurosurgical intensive care unit (NICU).

All patients were monitored and managed according to a standardized institutional protocol including hypothermia and other intensive care treatments. An intraventricular catheter or an intraparenchymatous microtransducer was used for the continuous measurement of intracranial pressure (ICP). Mild hypothermia (34–35 °C) was rapidly induced using cooling blankets (MTRE Advanced Technologies, Trevose, PA, USA) placed below and above the patients, which was maintained for as long as 3 days. The cooled patients were tracheotomized for ventilation, continuously sedated (chlorpromazine, 5–10 mg/h) and paralyzed (atracurium, 10–40 mg/h). During hypothermia, the systolic blood pressure of the patient was maintained above 90 mmHg, peripheral oxygen saturation greater than 95 %, ICP less than 25 mmHg, and cerebral perfusion pressure at a level of 50–70 mmHg. After a 3-day maintenance period, the patients were passively rewarmed to 36–37 °C at a rate no greater than 0.5 °C every 4 hours, carefully individualized by the patients’ ICP level. If the rebound of ICP was indicated, then a much slower rewarming rate (<0.5 °C every 8 hours) and the extensive use of other ICP-reducing measures were employed.

Intracranial hypertension during the whole hospitalization course was resolved progressively with a set of stepwise strategies including head elevation, ventilation control, sedation, hyperosmolar therapy, and ventricular drainage. If the raised ICP was resistant to these therapies, then decompressive craniotomy and/or further surgical operation were adopted. In addition, serum glucose, blood gases, and serum electrolytes were assessed regularly, with treatment provided for any abnormal findings. After hypothermia was discontinued, any patients with fever, defined as body temperature more than 38 °C, were examined for infectious or noninfectious causes and treated with ice packs around the neck and limbs, sponging the body with alcohol, use of paracetamol, or a combination.

### Biomarker determination

Blood samples (3–5 ml) were taken by the study investigators (JL and GG) using red top tubes at the time of admission (within 4 hours post injury) to the hospital and then each morning (6:00–8:00 am) for the first 5 days in the NICU. After collection, specimens were maintained at room temperature for 30–60 minutes and centrifuged for 10 minutes at 4000 rpm; sera were stored at −80 °C until analysis.

Serum GFAP concentration was measured using an enzyme-linked immunosorbent assay (ELISA) via a commercial kit according to the manufacturer’s protocol (Biovendor, Candler, NC, USA). Briefly, standards, quality controls (QC-Low and QC-High), and serum samples were incubated in microplate wells precoated with polyclonal anti-human GFAP antibody for 2 hours, followed by 1 hour incubation with a biotin-labeled monoclonal anti-human GFAP antibody solution and 1 hour incubation with a streptavidin–horseradish peroxidase conjugate. Between each step, the plate was thoroughly washed four times with washing buffer. After the last washing step, the remaining conjugate was allowed to react with the substrate solution for an additional 15 minutes. The reaction was stopped by the addition of an acidic solution, and the absorbance of the resulting yellow product was measured by reading the ELISA plate at 450 nm. The absorbance was proportional to the concentration of GFAP. A standard curve (0.25–25 ng/ml) was constructed by plotting absorbance values against concentrations of standards, and concentrations of unknown samples were determined using this standard curve. The lower limit for detection in the assay was 0.045 ng/ml. All concentrations below this limit of detection were reported as zero. The coefficients of variation for inter- and intra-assay variability were 5.7 % and 5.1 %, respectively. All samples were assayed in duplicate, and average results were used for analysis.

### Data collection and outcome assessment

Demographic and clinical data were collected from patient medical records including sex, age, and cause of injury, CT scan, admission GCS score, pupil reactions, and surgery. Initial head CT scans were analyzed and classified according to the criteria raised by Marshall and associates [19] to determine brain injury severity (Diffuse injury I: no visible intracranial pathology; Diffuse injury II: cisterns present with midline shift of 0–5 mm, no focal lesion of > 25 ml; Diffuse injury III: cistern compressed or absent with midline shift of 0–5 mm, no focal lesion of > 25 ml; Diffuse injury IV: midline shift of > 5 mm, no focal lesion of > 25 ml; Evacuated mass lesion: any lesion surgically evacuated; Non-evacuated mass lesion: lesion of > 25 ml, not surgically evacuated). Since the recent trial by Clifton et al. [20] reported possible differential effects of hypothermia among patients with surgical lesion compared with diffuse injury, the surgical lesion and diffuse injury groups were further defined based on the above CT classification in our study to examine the potential correlation between serum GFAP concentration and the pathological types of brain injury and the role of hypothermia.

Neurological outcome assessment was performed by a blinded investigator (CW) who was unaware of the patient's clinical history and biomarker data using the Glasgow Outcome Scale (GOS) at 6 months after injury with the use of a structured interview by direct patient contact or via telephone [21]. The GOS is a five-category scale used for assessing the neurological outcome after brain injury as follows: 1, death; 2, vegetative state—unable to interact with the environment; 3, severe disability—unable to live independently but able to follow commands; 4, moderate disability—capable of living independently but unable to return to work or school; and 5, good recovery—able to return to work or school. For statistical analyses, the outcome was further dichotomized in death (GOS 1) versus survival (GOS 2–5) and unfavorable (GOS 1–3) versus favorable (GOS 4–5).

### Statistical analysis

Basic descriptive statistics were used to describe the data, including standard measures of central tendency and dispersion for continuous data (mean, standard deviation (SD), median, and range) and frequencies and proportions for categorical variables. Comparisons were made using a chi-squared test, Fisher’s exact test, unpaired Student *t* test, or Wilcoxon rank-sum test, if appropriate. Significance was set at *P* < 0.05.

To analyze the association of serum GFAP levels during the first 6-day period and neurological outcomes at 6-month post injury, we use the longitudinal multiple linear regression models (also called cross-sectional time series) that controlled for confounding clinical parameters, which have the ability to provide efficient statistical power to analyze continuous outcomes (biomarker levels) that are measured at regular time intervals, which are the same for all subjects [22]. Serum GFAP levels were logarithmically transformed to achieve approximately normal distribution and treated as the dependent variable. Outcome group (i.e., death or unfavorable outcome, as defined above) and time of blood sampling were included in each model as independent variables. Additional variables, including age, sex, cause of injury, time from injury to admission, surgery, baseline GCS score, Marshall CT classification, and pupil reactions were evaluated as potential confounders and included in the models. These models allowed us to determine whether the injured patients from dichotomized outcome groups had differing serum GFAP levels over time.

Receiver operating characteristic (ROC) curve analysis was performed to determine the discriminatory characteristics of serum GFAP during the course from the day of admission to day 5. ROC curves were secondarily developed along with the respective diagnostic parameters of the coordinates of the curve (i.e., sensitivity, specificity, positive and negative predictive values), the area under the curve (AUC), and the optimal cutoff point for the best overall predictive ability (i.e., the sum of the sensitivity and the specificity for prediction of outcome was maximal). The AUC value was used to compare the ability of admission serum GFAP to predict 6-month lethal or unfavorable outcomes compared with the clinical predictors in the IMPACT core model (i.e., age, GCS score, or pupil reactions).

## Results

During the period from March 2011 to September 2014, there were 77 severe TBI patients deemed potentially eligible. Of these, 10 patients were not included in our study owing to lack of informed consent (n = 3), an age less than 18 years (n = 1), or loss to follow-up (n = 6). A final 67 patients were enrolled in this study. Clinical and demographic data of the study population are summarized in Table 1. The subjects ranged between 18 and 70 years of age (mean age: 37.2 ± 14.3 years), with the majority being males (n = 51, 76.1 %). Traffic accident was the most common cause of injury, accounting for 49.3 % of all subjects.

**Table 1:** Clinical and demographic characteristics of the 67 studied patients by outcome category at 6 months post injury

	Total (n = 67)	Death (GOS = 1)			Unfavorable outcome (GOS = 1–3)		
		Yes (n = 27)	No (n = 40)	*P* value	Yes (n = 35)	No (n = 32)	*P* value
Age (yrs, mean ± SD)	37.2 ± 14.3	42.0 ± 16.1	33.9 ± 12.0	0.039	40.0 ± 15.0	34.1 ± 12.9	0.503
Male, n (%)	51 (76.1)	21 (77.8)	30 (75.0)	0.794	28 (80.0)	23 (71.9)	0.436
Cause of injury, n (%)							
Traffic accident	33 (49.3)	13 (48.1)	20 (50.0)	0.968	17 (48.6)	16 (50.0)	0.997
Fall	26 (38.8)	11 (40.7)	15 (37.5)		14 (40.0)	12 (37.5)	
Violence	6 (8.9)	2 (7.4)	4 (10.0)		3 (8.6)	3 (9.4)	
Other	2 (2.9)	1 (3.7)	1 (2.5)		1 (2.9)	1 (3.1)	
Time after injury (hrs, mean ± SD)	2.6 ± 1.1	2.6 ± 1.2	2.6 ± 1.1	0.333	2.4 ± 1.2	2.9 ± 1.1	0.791
GCS, median (range)	6 (3–8)	5 (3–8)	7 (3–8)	0.006	5 (3–8)	7 (3–8)	0.001
GCS 3–5, n (%)	25	16 (59.3)	9 (22.5)	0.012	19 (54.3)	6 (18.8)	0.006
GCS 6–8, n (%)	42	11 (40.7)	31 (77.5)		16 (45.7)	26 (81.2)	
Pupil reactions, n (%)							
Both present	38	10 (37.0)	28 (41.8)	0.015	13 (37.1)	25 (78.1)	0.032
At least one unreactive pupil	29	17 (63.0)	12 (58.2)		22 (62.9)	7 (21.9)	
Marshall CT classification, n (%)							
Missing	3 (4.5)	1 (3.7)	2 (5)	0.641	1 (2.9)	2 (6.3)	0.406
Diffuse injury I	0 (0)	0 (0)	0 (0)		0 (0)	0 (0)	
Diffuse injury II	7 (10.4)	3 (11.1)	4 (10)		4 (11.4)	3 (9.4)	
Diffuse injury III	11 (16.4)	6 (22.2)	5 (12.5)		6 (17.1)	5 (15.6)	
Diffuse injury IV	13 (19.4)	6 (22.2)	7 (17.5)		10 (28.6)	3 (9.4)	
Evacuated mass lesion	30 (44.8)	11 (40.7)	19 (47.5)		13 (37.1)	17 (53.1)	
Non-evacuated mass lesion	3 (4.5)	0 (0)	3 (7.5)		1 (2.9)	2 (6.3)	
Neurosurgery, n (%)							
Yes	59 (88.1)	25 (92.6)	34 (85.0)	0.347	33 (94.3)	26 (81.3)	0.100
No	8 (11.9)	2 (7.4)	6 (15.0)		2 (5.7)	6 (18.7)	
Duration of neurosurgery, (hrs, mean ± SD)^a^	2.6 ± 1.3	3.1 ± 1.2	2.3 ± 1.3	0.704	3.2 ± 1.2	1.9 ± 1.0	0.216

*GOS* Glasgow Outcome Scale, *yrs* years, *SD* standard deviation, *hrs* hours, *GCS* Glasgow Coma Scale, *CT* computed tomography

^a^Documented in 59 of 67 patients

On average, patients were admitted to hospital within 2.6 hours (SD: 1.1, range: 0.5–4) of injury, with a median admission GCS score of 6 (range: 3–8). Except for three subjects with missing CT images, 31 of the 64 patients presented with diffuse injury (Diffuse injury II to IV) and 33 with focal mass lesion (Evacuated mass lesion, n = 30; Non-evacuated mass lesion, n = 3) according to the Marshall classification. For 59 cases, neurosurgical operation was performed and lasted for a mean (SD) of 2.6 (1.3) hours (range: 0.5–5). ICP was continuously monitored by intraventricular (n = 51) or intraparenchymal (n = 16) routes and the value was at least once above 25 mmHg in 34 patients (nine patients with unavailable data). The neurological outcome was assessed by telephone or regular clinical visit at 6 months later, when 40 of 67 patients were alive, of whom 32 had a favorable outcome (GOS 4–5). Patients in the lethal or unfavorable outcome groups were more likely to be older and have a lower admission GCS score, and abnormal pupils. Otherwise, there were no significant differences in terms of sex, cause of trauma, time from injury to admission, Marshall CT classification, neurosurgical intervention, and duration of surgery between outcome groups.

Serial blood samples were collected on admission (D0), days 1 (D1), 2 (D2), 3 (D3), 4 (D4), and 5 (D5) post injury for the enrolled patients. Nine blood specimens were not obtained from D0 to D3 (two at D0, four at D1, three at D2, and two at D3). Four patients died during the first 5 days, and thus did not have blood drawn at either D5 (n = 3) or at both D4 and D5 (n = 1). The remaining 54 (80.6 %) patients had blood samples collected at all time points. In addition, blood samples were obtained from 135 healthy blood donors (88 males and 47 females; mean age of 39.2 ± 15.3 years; range: 18–65).

Medians (interquartile range; IQR) for serum GFAP concentrations in the severe TBI patients from D0 to D5 and healthy subjects were 1.924 (0.891–3.126) ng/ml, 1.079 (0.468–1.880) ng/ml, 0.790 (0.402–1.228) ng/ml, 0.751 (0.438–1.206) ng/ml, 1.006 (0.534–1.529) ng/ml, 0.649 (0.284–0.982) ng/ml, and 0 (0–0) ng/ml, respectively. For healthy individuals, only 12 of 135 serum samples had detectable GFAP (range: 0.048–0.076 ng/ml; median: 0.058 ng/ml). The differences in GFAP levels between TBI patients and controls were highly significant at all time intervals (*P* < 0.001 for each comparison).

**Fig. 1:**
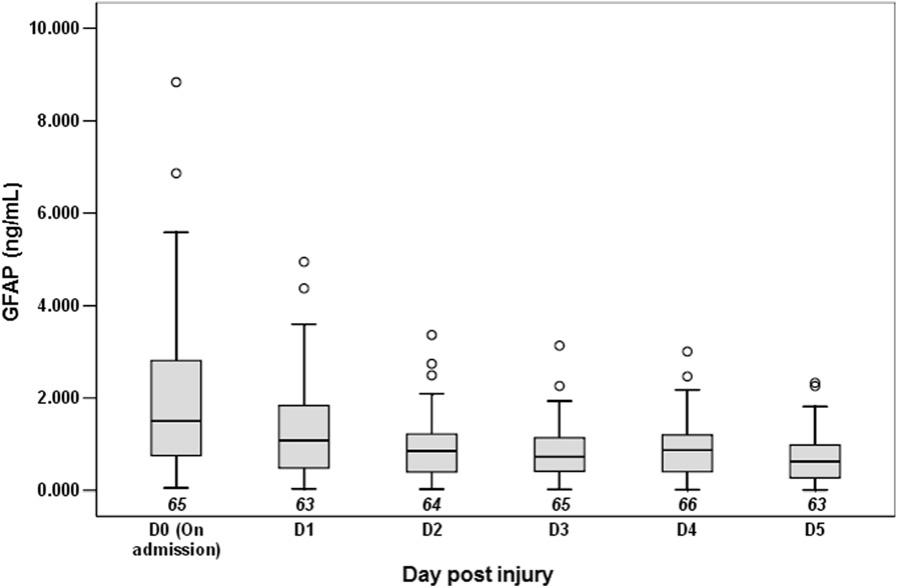
Serum GFAP levels by the overall population during the first 6 days post injury. D0, D1, D2, D3, D4, and D5 represent the admission day and days 1–5 post injury, respectively. Box and whisker plots show median, interquartile range (IQR), and values within ± 1.5 of IQR. Outliers are plotted as *open circles*. The number under each box is the number of samples available for analysis. Hypothermia was induced in patients when admitted into the NICU, and was maintained for the first 3 days then terminated with slow rewarming. *GFAP* glial fibrillary acidic protein, *NICU* neurosurgical intensive care unit

**Fig. 2:**
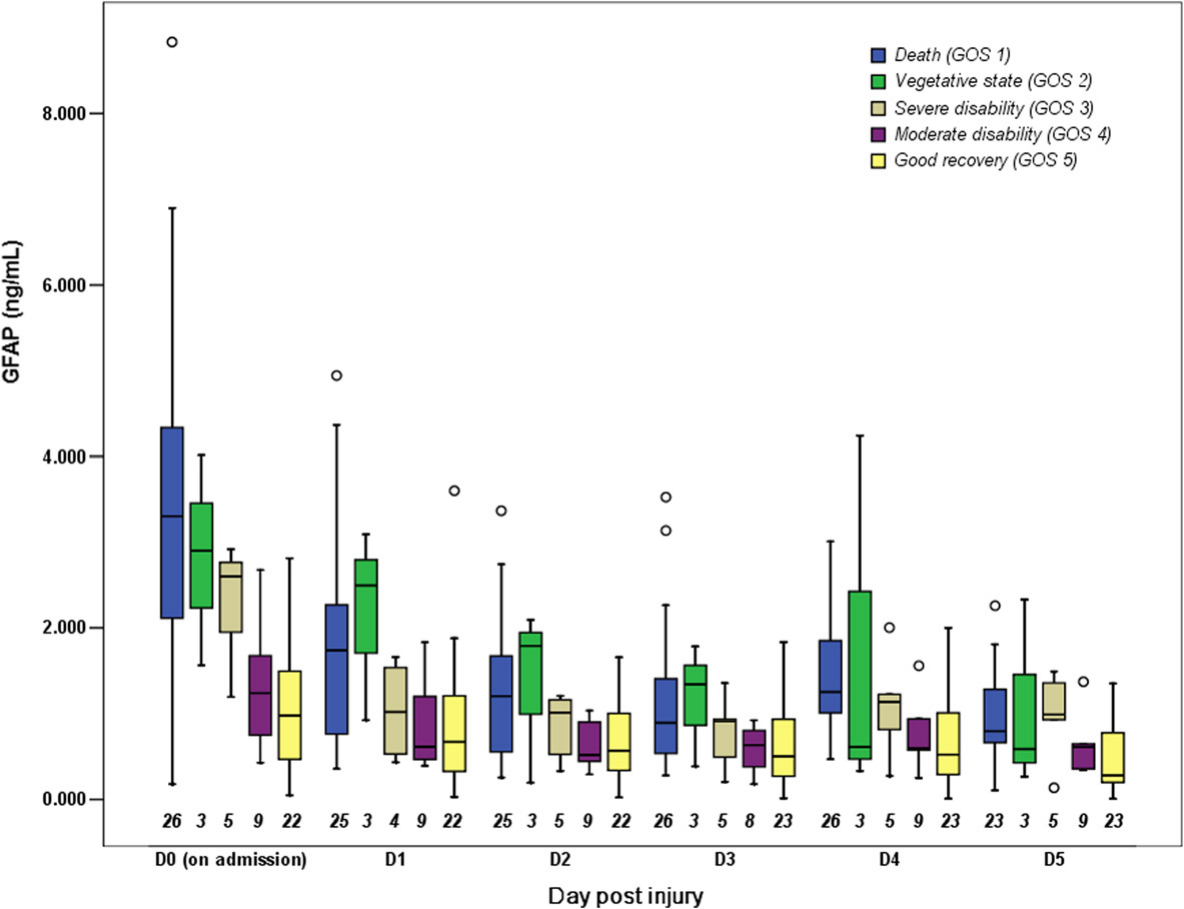
Serum GFAP levels by each outcome group over the first 6 days post injury, shown as box plots. D0, D1, D2, D3, D4, and D5 represent the admission day and days 1–5 post injury, respectively. Box and whisker plots show median, interquartile range (IQR), and values within ± 1.5 of IQR. Outliers are plotted as *open circles*. The number under each box is the number of samples available for analysis. Hypothermia was induced in patients when admitted into the NICU, and was maintained for the first 3 days then terminated with slow rewarming. *GFAP* glial fibrillary acidic protein, *NICU* neurosurgical intensive care unit

**Fig. 3:**
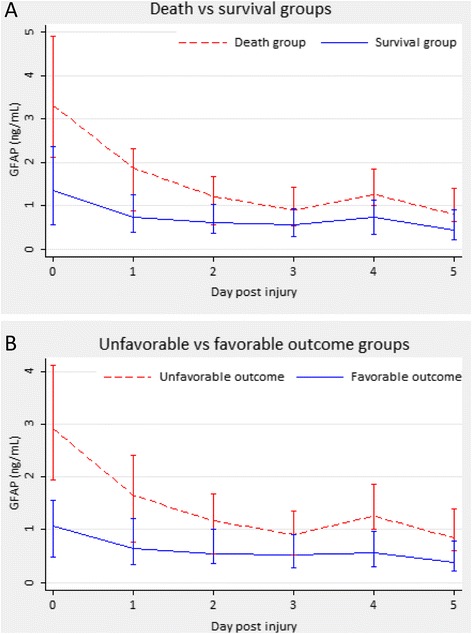
Median serum GFAP levels by categorized outcome groups. **a** Death versus survival. **b** Unfavorable versus favorable outcome. Error bars show interquartile ranges. *GFAP* glial fibrillary acidic protein

The time course of serum GFAP levels by the overall population and each outcome category is presented in Figs. 1 and 2. The serum GFAP value gradually decreased over the first 3 days until the rebound on day 4, when hypothermia was discontinued with slow rewarming. Thirty-eight of the 64 (59.4 %) patients had elevated serum GFAP level on day 4 compared with day 3 (24 of 33 with unfavorable outcome versus 14 of 31 with favorable outcome, respectively, *P* = 0.016). Serum GFAP levels over the study period were significantly higher in the patients who died within 6 months after injury than in those who were alive or had favorable outcome (Fig. 3). These differences remained statistically significant after controlling for covariables, including age, sex, cause of injury, time from injury to admission, surgery, baseline GCS score, Marshall CT classification, and pupil reactions. Among these covariables, baseline GCS score and pupil reactions were consistently significant in the models both for death/survival or unfavorable/favorable outcomes (*P* < 0.05 for both covariates in the final models).

With regard to the pathological types of TBI based on the Marshall classification, 31 patients were diagnosed with diffuse injury (Diffuse injury II–IV) and 30 patients with surgical lesions (Evacuated mass lesion) on the initial head CT scan. Table 2 describes the characteristics of both groups. There was a well-matched baseline between patients with diffuse injury and surgical lesion, with no differences between groups regarding the demographic, clinical characteristics, and 6-month outcome. Serum GFAP concentrations on day 0 were significantly higher in patients with surgical lesion compared with patients with diffuse injury (median (IQR), 2.776 (1.538–3.942) ng/ml vs. 1.151 (0.459–1.847) ng/ml; *P* < 0.001). On day 4 after hypothermia treatment, the GFAP levels decreased to 1.035 (IQR, 0.882–1.663) ng/ml in the surgical lesion group and to 0.868 (IQR, 0.359–1.265) ng/ml in the diffuse injury group. With the logarithmically transformed data, there was a significant difference of reduction in serum GFAP between groups after controlling for the baseline concentration (*P* = 0.037).

**Table 2:** Patient characteristics between diffuse injury and surgical lesion group

	Diffuse injury	Surgical lesion	*P* value
	(n = 31)	(n = 30)	
Age (yrs, mean ± SD)	35.1 ± 14.3	40.6 ± 14.9	0.917
Male, n (%)	24 (77.4)	23 (76.7)	0.944
GCS, median (range)	6 (3–8)	6 (3–8)	0.071
GCS 3–5, n (%)	10 (32.3)	14 (46.7)	0.300
GCS 6–8, n (%)	21 (67.7)	16 (53.3)	
Pupil reactions, n (%)			
Both present	20 (64.5)	13 (43.3)	0.126
At least one unreactive pupil	11 (35.5)	17 (56.7)	
6-month outcome, n (%)			
Death/alive	15 (48.4)/16 (51.6)	11 (36.7)/19 (63.3)	0.440
Unfavorable/favorable	20 (64.5)/11 (35.5)	13 (43.3)/17 (56.7)	0.202

*Yrs* years, *SD* standard deviation, *GCS* Glasgow Coma Scale

**Fig. 4:**
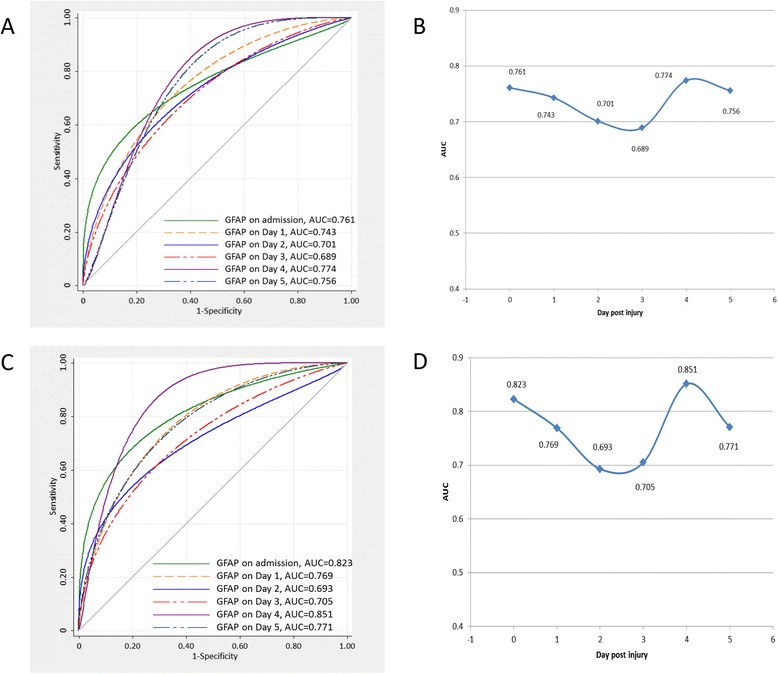
Receiver operating curves (ROC) for serum GFAP from the admission day to day 5 post injury and prediction of death (**a**) and unfavorable outcome (**c**). Each day has a representative curve. The area under the curve (AUC) over time was also plotted (**b** and **d**). *GFAP* glial fibrillary acidic protein

Figure 4 shows the ROC analyses of serum levels of GFAP on days 0, 1, 2, 3, 4, and 5 post injury. AUC data on each day are also plotted. The AUC values in serum GFAP were significantly greater than 0.5 at each time point (*P* < 0.05), which ranged from 0.689 to 0.774 for death and from 0.693 to 0.851 for unfavorable outcome (Fig. 4a and c). Consistent with the change in serum levels of GFAP in the first 6 days, AUC values also showed a similar decrease from D0 to D3 and then a further increase on D4 (Fig. 4b and d). Interestingly, serum GFAP on D4 (the day after hypothermia) had much higher AUC values than for D0, which may suggest the potential clinical utility of delayed determination of biomarkers after a certain intervention. The predictive ability of serum GFAP at the time of admission was good, with AUCs of 0.761 (95 % CI, 0.606–0.917) for death and 0.823 (95 % CI, 0.700–0.947) for unfavorable outcome. These values were higher compared with the clinical variables including age, GCS score, and pupil reactions in both predictions of death and unfavorable outcome (Table 3).

**Table 3:** Predictive values of admission serum GFAP and clinical variables for death and unfavorable outcome at 6 months of injury

	Death (GOS = 1)						Unfavorable outcome (GOS = 1–3)					
Variable	Cutoff	Sensitivity	Specificity	PPV	NPV	AUC (95 % CI)	Cutoff	Sensitivity	Specificity	PPV	NPV	AUC (95 % CI)
GFAP (ng/ml)	1.690	84.6	69.2	64.7	87.1	0.761 (0.606, 0.917)	1.559	85.3	77.4	80.6	82.8	0.823 (0.700, 0.947)
Age (years)	44.5	44.4	82.5	63.2	68.8	0.592 (0.503, 0.681)	39.5	45.7	65.6	59.3	52.5	0.617 (0.482, 0.751)
GCS score	4.5	90.0	48.1	72.0	76.5	0.742 (0.616, 0.868)	5.5	81.3	54.3	61.9	76.0	0.751 (0.620, 0.862)
Pupil reactions	Absent	70.0	63.0	73.7	58.6	0.665 (0.530, 0.800)	Absent	78.1	62.9	65.8	75.9	0.705 (0.578, 0.832)

The (optimal) cutoff values were determined using receiver operating characteristic curve under the condition of equal costs of misclassification of cases and non-cases; i.e., the sum of the sensitivity and the specificity to predict the chosen outcome category was maximal

*GFAP* glial fibrillary acidic protein, *GOS* Glasgow Outcome Scale, *PPV* positive predictive value, *NPV* negative predictive value, *AUC* area under the curve, *CI* confidence interval, *GCS* Glasgow Coma Scale

The optimal cutoff point and associated diagnostic characteristics for serum GFAP levels on admission were calculated from the coordinates of the ROC curve for the best overall predictive performance of outcomes. Using the cutoff value of 1.690 ng/ml, serum GFAP on admission had a sensitivity of 84.6 % and specificity of 69.2 % for predicting death, with a positive predictive value (PPV) of 64.7 % and a negative predictive value (NPV) of 87.1 %. For prediction of unfavorable outcome at 6 months post injury, admission GFAP (the optimal cutoff value, 1.559 ng/ml) had a sensitivity of 85.3 %, specificity of 77.4 %, PPV of 80.6 % and NPV of 82.8 % (Table 3).

## Discussion

Improving the prognosis for patients with acute TBI is critical [23] as TBI remains a devastating disease associated with considerable mortality and morbidity and a huge burden to society, and there are no interventions that show substantial efficacy despite of hundreds of trials [24, 25]. Although hypothermia shows the most clinical promise for improving patient outcomes, the optimal clinical protocols remain unclear [26]. The use of biomarkers may help to detect, track cellular damage and cerebral protection in the injured brain, and to balance the risks and benefits of therapeutic interventions.

Biomarkers can also be used to provide outcome prediction and risk triage. Current assessment tools include clinical characteristics (e.g., age, sex, reaction of the pupils, GCS score, and secondary insults), radiological data, and electroencephalography, which provide some predictive information. However, there are inherent limitations in these procedures. Clinical examinations are usually subjective and are often confounded by medications such as sedation, intubation/tracheostomy, and muscle relaxants, making them less reliable in the setting of the state-of-the-art critical management including hypothermia and early sedation. Head CT scanning, magnetic resonance imaging, or eletroencephalography often require specialized equipment or technical/interpretive expertise that may not be available at resource-limited medical centers. Further, they do not offer adequate information on the biochemical changes in the brain [27]. Thus, identification of valid neurobiochemical markers of brain injury that serve as surrogates of the presence, evolution, and outcomes of brain injury is key to the research and treatment of TBI patients.

GFAP, a brain-specific protein that functions as the major integral component of the cytoskeleton of astrocytes, has been recently reported as a blood biomarker. After brain damage, GFAP is released from degenerating brain cells into the surrounding interstitial fluid and appear in the peripheral blood, likely via disruptions in the blood–brain barrier [28]. Elevated serum GFAP can provide diagnostic and prognostic information in a range of neurological diseases including neurodegenerative disorders [29], stroke [30], and severe TBI [31]. In line with previous reports, our study in a cohort of 67 patients with severe TBI demonstrated that elevated serum GFAP on admission and during the subsequent 5 days had a strong correlation with death and unfavorable outcome at 6 months post injury, which was independent of baseline and clinical characteristics of brain injury. Additionally, our study also indicates that the predictive accuracy of admission GFAP was greater than those of clinical variables including age, GCS score, and pupil reactions, which may provide further evidence about the utility of GFAP in the care of TBI patients.

Induction of mild hypothermia immediately after brain injury can reduce cerebral metabolism and oxygen consumption, diminish cytotoxic edema, stabilize the blood–brain barrier, and improve histological and functional outcomes and survival in numerous animal and clinical studies [32]. Mild hypothermia is now an accepted treatment for anoxic brain injury following cardiac arrest and neonatal hypoxic-ischemic encephalopathy [33, 34]. Although recent multicenter trials have reported negative results for treatment of severe TBI with hypothermia, numerous single-center studies and several later meta-analyses support its neuroprotective actions, especially when maintained for longer than 48 hours [20, 35, 36].

Apart from the insensitive methodology or potential biases such as center effects in these multicenter trials, the most likely explanation for these conflicting results is the different protocols of hypothermia therapy used clinically or the heterogeneous types of TBI patients enrolled [37]. For example, in a recent large clinical trial by Clifton and colleagues [20], a post hoc subgroup analysis (28 patients were allowed for analysis, 15 of whom treated by hypothermia) suggested a specific beneficial effect of hypothermia in patients with surgically evacuated hematoma. Yokobori et al. also reported that hypothermia could reduce histological and biochemical injury to neurons and glia in a rat model of acute hematoma of severe TBI [38]. In the present study, although the long-term neurological outcomes did not show any differences between patients with surgical lesion and diffuse injury, the change in serum GFAP before and after hypothermia was significantly greater among patients with surgical lesion, suggesting a more profound neuroprotective effect of hypothermia among this group of patients. Thus, supported by the above evidence and our present data, a further study, targeted specifically on the type of surgical lesion, is warranted to investigate the different effect of hypothermia in the setting of TBI.

We found a persistent reduction of serum GFAP level during the first few days after TBI in hypothermia-treated patients in both the favorable and unfavorable outcome groups. However, as we did not have a normothermia-treated control group, this reduction may also be attributed to altered permeability of the blood–brain barrier due to hypothermia [39], or the natural temporal alterations of serum GFAP in TBI patients, rather than solely to a neuroprotective effect of hypothermia. Future studies are required to examine the effect of hypothermia on serum concentrations of biochemical markers and the correlation of the altered levels and long-term neurological outcomes. Our ongoing LTH-1 hypothermia trial and collection of biofluid samples of patients has the potential to address this issue in the future [40].

The marked increase of serum GFAP levels at the conclusion of the 3-day maintenance period of hypothermia was observed in 38 of 64 patients, 24 of whom had unfavorable outcome at 6 months post injury, suggesting that continued secondary injury in these cases that was suppressed by cooling therapy or the possible evidence of rewarming injury. Thus, these 38 patients may benefit from alterations in hypothermia protocols including longer cooling or more advanced rewarming procedures. The optimal duration and rewarming rate of hypothermia for severe TBI are extremely important issues to be addressed, as numerous studies report that effective hypothermia treatment in TBI patients requires early initiation, with suitable length of cooling and steady rewarming [41]. As there are no reliable surrogate markers for monitoring the pathophysiology of the injured brain, gauging response to treatment, and comparing the effects of various hypothermia protocols, it is possible that circulating injury markers like GFAP will help to bridge this gap.

The cutoff value we found for admission serum GFAP to predict unfavorable outcome at 6 months was 1.559 ng/ml, similar to the 1.5 ng/ml reported by Vos and colleagues [9, 31]. However, a wide range of cutoff points from 1.5 to 6.98 ng/ml have been reported, leading to different sensitivities and specificities for this protein, which limits the utility of GFAP as a reliable predictor in clinical practice [14, 16, 42]. To address this problem, further validation studies in a larger sample of participants is required, using standardized assays with well-established, consistent, and clinically interpretable thresholds for GFAP alterations.

There are several limitations of our study. First, we enrolled a relatively limited sample of patients with severe TBI, a disease with extremely intrinsic heterogeneity. With this small sample size and the observational study design, we failed to detect significant differences in long-term outcomes between patients with surgical lesions and diffuse injury, and observed inconsistency between biomarker changes and long-term outcomes. Second, it is unclear whether the ELISA kit used to measure the serum GFAP cross-reacts with GFAP breakdown products (GFAP-BDPs). A study in 108 samples assessing the utility of serum GFAP-BDPs found a detectable increase in the blood within an hour of head injury, which was associated with injury severity and CT lesions [43]. This may alter the final interpretation for the predictive ability of the intact GFAP in our study. Third, serum biomarker alterations may not completely reflect the biochemical changes in the injured brain as they can be influenced by various factors including the integrity of the blood–brain barrier or other factors that regulate the release of proteins into the peripheral circulation in the settings of brain injury and hypothermia. However, a high correlation between GFAP concentration in serum and cerebrospinal fluid (CSF) was reported [44]. Further, serial sampling of CSF is often contraindicated in patients with severe TBI, especially with obvious intracranial hypertension. Therefore, serum analysis may have more clinical advantages than CSF measurement and should be considered in future clinical studies. Fourth, several technical variables should be considered, such as time to process the blood sample, effect of temperature change, and methodology of measurement, which can affect the stability and reliability of results. Fifth, a control group of severe TBI patients without hypothermia treatment was not included in our study. Thus, we could not determine the possible effect of hypothermia on serum GFAP levels for TBI patients. Finally, some patient serum specimens were unable to be obtained on some days. However, the effect of missing samples on the whole analysis may be minimal owing to the sensitive statistical methodology we used.

## Conclusions

Our study suggests that serum GFAP might be a useful biochemical marker for severe TBI patients. Elevated serum GFAP on admission and over a range of days post injury was a strong predictor for death and unfavorable outcome at 6 months. Because of increasing clinical evidence that GFAP is highly brain-specific and shows diagnostic, prognostic, and even theranostic utility for clinicians, further studies should be encouraged to fully elucidate the basic mechanisms and validate the potential role of GFAP as a biomarker for TBI.

## Key messages

- Serum GFAP levels on admission and the first 5 days were elevated among patients with severe TBI compared with controls.
- Elevated serum GFAP levels over the first 6 days had predictive power for neurological outcome at 6 months post injury.
- Admission GFAP levels had greater predictive ability than clinical characteristics including age, GCS score, and pupil reactions.

## Abbreviations

AUC: area under the curve
CSF: cerebrospinal fluid
CT: computed tomography
ED: emergency department
ELISA: enzyme-linked immunosorbent assay
EMS: emergency medical services
GCS: Glasgow Coma Scale
GFAP: glial fibrillary acidic protein
GFAP-BDPs: GFAP breakdown products
GOS: Glasgow Outcome Scale
ICP: intracranial pressure
IQR: interquartile range
NICU: neurosurgical intensive care unit
NPV: negative predictive value
PPV: positive predictive value
ROC: receiver operating characteristic
SD: standard deviation
TBI: traumatic brain injury
